# JANUS: A hypothesis-driven Bayesian approach for understanding edge formation in attributed multigraphs

**DOI:** 10.1007/s41109-017-0036-1

**Published:** 2017-06-24

**Authors:** Lisette Espín-Noboa, Florian Lemmerich, Markus Strohmaier, Philipp Singer

**Affiliations:** 1 GESIS - Leibniz Institute for the Social Sciences, Unter Sachsenhausen 6-8, Cologne, 50667 Germany; 2 University of Koblenz-Landau, Koblenz, 56070 Germany

**Keywords:** Edge formation, Bayesian inference, Attributed multigraphs, Multiplex, HypTrails

## Abstract

Understanding edge formation represents a key question in network analysis. Various approaches have been postulated across disciplines ranging from network growth models to statistical (regression) methods. In this work, we extend this existing arsenal of methods with JANUS, a hypothesis-driven Bayesian approach that allows to intuitively compare hypotheses about edge formation in multigraphs. We model the multiplicity of edges using a simple categorical model and propose to express hypotheses as priors encoding our belief about parameters. Using Bayesian model comparison techniques, we compare the relative plausibility of hypotheses which might be motivated by previous theories about edge formation based on popularity or similarity. We demonstrate the utility of our approach on synthetic and empirical data. JANUS is relevant for researchers interested in studying mechanisms explaining edge formation in networks from both empirical and methodological perspectives.

## Introduction

Understanding edge formation in networks is a key interest of our research community. For example, social scientists are frequently interested in studying relations between entities within social networks, e.g., how social friendship ties form between actors and explain them based on attributes such as a person’s gender, race, political affiliation or age in the network (Sampson 1968). Similarly, the complex networks community suggests a set of generative network models aiming at explaining the formation of edges focusing on the two core principles of *popularity* and *similarity* (Papadopoulos et al. 2012). Thus, a series of approaches to study edge formation have emerged including statistical (regression) tools (Krackhardt 1988; Snijders et al. 1995) and model-based approaches (Snijders 2011; Papadopoulos et al. 2012; Karrer and Newman 2011) specifically established in the physics and complex networks communities. Other disciplines such as the computer sciences, biomedical sciences or political sciences use these tools to answer empirical questions; e.g., co-authorship networks (Martin et al. 2013), wireless networks of biomedical sensors (Schwiebert et al. 2001), or community structures of political blogs (Adamic and Glance 2005).

**Fig. 1:**
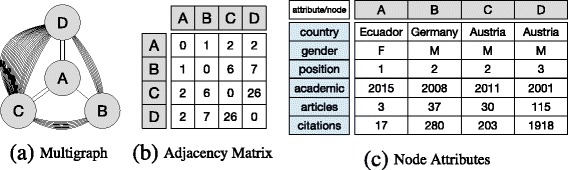
Example: This example illustrates an unweighted attributed multigraph. **a** Shows a multigraph where nodes represent academic researchers, and edges scientific articles in which they have collaborated together. **b** Shows the adjacency matrix of the graph, where every cell represents the total number of edges between two nodes. **c** Decodes some attribute values per node. For instance, node D shows information about an *Austrian* researcher who started *his* academic career in *2001*. One main objective of JANUS is to compare the plausibility of mechanisms derived from attributes for explaining the formation of edges in the graph. For example, here, a hypothesis that researchers have more collaborations if they are from the same country might be more plausible than one that postulates that the multiplicity of edges can be explained based on the relative popularity of authors

**Problem illustration** Consider for example the network depicted in Fig. 1. Here, nodes represent authors, and (multiple) edges between them refer to co-authored scientific articles. Node attributes provide additional information on the authors, e.g., their home country and gender. In this setting, an exemplary research question could be: “Can co-authorship be better explained by a mechanism that assumes more collaborations between authors from the *same country* or by a mechanism that assumes more collaborations between authors with the *same gender*?”. These and similar questions motivate the main objective of this work, which is to provide a Bayesian approach for understanding how edges emerge in networks based on some characteristics of the nodes or dyads.

While several methods for tackling such questions have been proposed, they come with certain limitations. For example, statistical regression methods based on QAP (Hubert and Schultz 1976) or mixed-effects models (Shah and Sinha 1989) do not scale to large-scale data and results are difficult to interpret. For network growth models (Papadopoulos et al. 2012), it is necessary to find the appropriate model for a given hypothesis about edge formation and thus, it is often not trivial to intuitively compare competing hypotheses. Consequently, we want to extend the methodological toolbox for studying edge formation in networks by proposing a first step towards a hypothesis-driven generative Bayesian framework.

**Approach and methods** We focus on understanding edge formation in attributed multigraphs. We are interested in modeling and understanding the multiplicity of edges based on additional network information, i.e., given attributes for the nodes or dyads in the network. Our approach follows a generative storyline. First, we define the model that can characterize the edge formation at interest. We focus on the simple categorical model, from which edges are independently drawn from. Motivated by previous work on sequential data (Singer et al. 2015), the core idea of our approach is to specify generative hypotheses about how edges emerge in a network. These hypotheses might be motivated by previous theories such as popularity or similarity (Papadopoulos et al. 2012)—e.g., for Fig. 1 we could hypothesize that authors are more likely to collaborate with each other if they are from the same country. Technically, we elicit these types of hypotheses as beliefs in parameters of the underlying categorical model and encode and integrate them as priors into the Bayesian framework. Using Bayes factors with marginal likelihood estimations allows us to compare the relative plausibility of expressed hypotheses as they are specifically sensitive to the priors. The final output is a ranking of hypotheses based on their plausibility given the data.

**Contributions** The main contributions of this work are:

1. We present a first step towards a Bayesian approach for comparing generative hypotheses about edge formation in networks.
2. We provide simple categorical models based on local and global scenarios allowing the comparison of hypotheses for multigraphs.
3. We show that JANUS can be easily extended to dyad-attributed multigraphs when multiplex networks are provided.
4. We demonstrate the applicability and plausibility of JANUS based on experiments on synthetic and empirical data, as well as by comparing it to the state-of-the-art QAP.
5. We make an implementation of this approach openly available on the Web (Espín-Noboa 2016).

**Structure** This paper is structured as follows: First, we start with an overview of some existing research on modeling and understanding edge formation in networks in Section “Related work”. We present some background knowledge required in this work in Section “Background” to then explain step-by-step JANUS in Section “Approach”. Next, we show JANUS in action and the interpretation of results, by running four different experiments on synthetic and empirical data in Section “Experiments”. In Section “Discussion” we suggest a fair comparison of JANUS with the Quadratic Assignment Procedure (QAP) for testing hypotheses on dyadic data. We also highlight some important caveats for further improvements. Finally, we conclude in Section “Conclusions” by summarizing the contributions of our work.

## Related work

We provide a broad overview of research on modeling and understanding edge formation in networks; i.e., *edge formation models* and *hypothesis testing on networks*.

**Edge formation models** A variety of models explaining underlying mechanisms of *network formation* have been proposed. Here, we focus on models explaining linkage between dyads beyond structure by incorporating node attribute information. Prominently, the *stochastic blockmodel* (Karrer and Newman 2011) aims at producing and explaining communities by accounting for node correlation based on attributes. The *attributed graph* (Pfeiffer III et al. 2014) models network structure and node attributes by learning the attribute correlations in the observed network. Furthermore, the *multiplicative attributed graph* (Kim and Leskovec 2011) takes into account attribute information from nodes to model network structure. This model defines the probability of an edge as the product of individual attribute link formation affinities. *Exponential random graph models* (Robins et al. 2007) (also called the *p* ^∗^ class of models) represent graph distributions with an exponential linear model that uses feature-structure counts such as reciprocity, k-stars and k-paths. In this line of research, *p1 models* (Holland and Leinhardt 1981) consider expansiveness (sender) and popularity (receiver) as fixed effects associated with unique nodes in the network (Goldenberg et al. 2010) in contrast to the *p2 models* (Robins et al. 2007) which account for random effects and assume dyadic independence conditionally to node-level attributes. While many of these works focus on binary relationships, (Xiang et al. 2010) proposes an unsupervised model to estimate continuous-valued relationship strength for links from interaction activity and user similarity in social networks. Recently, the work in (Kleineberg et al. 2016) has shown that connections in one layer of a multiplex can be accurately predicted by utilizing the hyperbolic distances between nodes from another layer in a hidden geometric space.

**Hypothesis testing on networks** Previous works have implemented different techniques to test hypotheses about network structure. For instance, the work in (Moreno and Neville 2013) proposes an algorithm to determine whether two observed networks are significantly different. Another branch of research has specifically focused on dyadic relationships utilizing regression methods accounting for interdependencies in network data. Here, we find *Multiple Regression Quadratic Assignment Procedure* (MRQAP) (Krackhardt 1988) and its predecessor QAP (Hubert and Schultz 1976) which permute nodes in such a way that the network structure is kept intact; this allows to test for significance of effects. *Mixed-effects models* (Shah and Sinha 1989) add random effects to the models allowing for variation to mitigate non-independence between responses (edges) from the same subject (nodes) (Winter 2013). Based on the *quasi essential graph* the work in (Nguyen 2012) proposes to compare two graphs (i.e., Bayesian networks) by testing and comparing multiple hypotheses on their edges. Recently, *generalized hypergeometric ensembles* (Casiraghi et al. 2016) have been proposed as a framework for model selection and statistical hypothesis testing of finite, directed and weighted networks that allow to encode several topological patterns such as block models where homophily plays an important role in linkage decision. In contrast to our work, neither of these approaches is based on Bayesian hypothesis testing, which avoids some fundamental issues of classic frequentist statistics.

## Background

In this paper, we focus on both *node-attributed* and *dyad-attributed* multigraphs with *unweighted edges without own identity*. That means, each pair of nodes or dyad can be connected by multiple indistinguishable edges, and there are features for the individual nodes or dyads available.

**Node-attributed multigraphs** We formally define this as: Let *G*=(*V,E*,*F*) be an unweighted attributed multigraph with *V*=(*v*_1_,…,*v*_*n*_) being a list of nodes, *E*={(*v*_*i*_,*v*_*j*_)}∈*V*×*V* a multiset of either directed or undirected edges, and a set of feature vectors *F*=(*f*_1_,…,*f*_*n*_). Each feature vector *f*_*i*_=(*f*_*i*_[1],...,*f*_*i*_[*c*])^*T*^ maps a node *v*_*i*_ to *c* (numeric or categorical) attribute values. The graph structure is captured by an adjacency matrix *M*_*n*×*n*_=(*m*_*ij*_), where *m*_*ij*_ is the multiplicity of edge (*v*_*i*_,*v*_*j*_) in *E* (i.e., number of edges between nodes *v*_*i*_ and *v*_*j*_). By definition, the total number of multiedges is $l=|E|=\sum _{ij} m_{ij}$.

Figure 1 a shows an example unweighted attributed multigraph: nodes represent authors, and undirected edges represent co-authorship in scientific articles. The adjacency matrix of this graph—counting for multiplicity of edges—is shown in Fig. 1 b. Feature vectors (node attributes) are described in Fig. 1 c. Thus, for this particular case, we account for *n*=4 nodes, *l*=44 multiedges, and *c*=6 attributes.

**Dyad-attributed networks** As an alternative to attributed nodes, we also consider multigraphs, in which each dyad (pair of nodes) is associated with a set of features $\hat F = (\hat f_{11}, \ldots, \hat f_{nn})$. Each feature vector $\hat f_{ij} = (\hat f_{ij}[1],..., \hat f_{ij}[c])^{T}$ maps the pair of node (*v*_*i*_, *v*_*j*_) to *c* (numeric or categorical) attribute values. The values of each feature can be represented in a separate *n*×*n* matrix. As an important special case of dyad-attributed networks, we study *multiplex networks*. In these networks, all dyad features are integer-valued. Thus, each feature can be interpreted as (or can be derived from) a separate multigraph over the same set of nodes. In our setting, the main idea is then to try and explain the occurrence of a multiset of edges *E* in one multigraph *G* with nodes *V* by using other multigraphs $\hat {G}$ on the same node set.

**Bayesian hypothesis testing** Our approach compares hypotheses on edge formation based on techniques from Bayesian hypothesis testing (Kruschke 2014; Singer et al. 2015). The elementary Bayes’ theorem states for parameters *θ*, given data *D* and a hypothesis *H* that:

1 $$ \overbrace{P(\theta| D, H)}^{\text{posterior}} = \frac{\overbrace{P(D | \theta, H)}^{\text{likelihood}}\overbrace{P(\theta|H)}^{\text{prior}}}{\underbrace{P(D|H)}_{\text{marginal likelihood}}}  $$

As observed data *D*, we use the adjacency matrix *M*, which encodes edge counts. *θ* refers to the model parameters, which in our scenario correspond to the probabilities of individual edges. *H* denotes a hypothesis under investigation. The *likelihood* describes, how likely we observe data *D* given parameters *θ* and a hypothesis *H*. The *prior* is the distribution of parameters we believe in before seeing the data; in other words, the prior encodes our hypothesis *H*. The *posterior* represents an adjusted distribution of parameters after we observe *D*. Finally, the *marginal likelihood* (also called *evidence*) represents the probability of the data *D* given a hypothesis *H*.

In our approach, we exploit the sensitivity of the marginal likelihood on the prior to compare and rank different hypotheses: more plausible hypotheses imply higher evidence for data *D*. Formally, *Bayes Factors* can be employed for comparing two hypotheses. These are computed as the ratio between the respective marginal likelihood scores. The strength of a Bayes factor can be judged using available interpretation tables (Kass and Raftery 1995). While in many cases determining the marginal likelihood is computationally challenging and requires approximate solutions, we can rely on exact and fast-to-compute solutions in the models employed in this paper.

## Approach

In this section, we describe the main steps towards a hypothesis-driven Bayesian approach for understanding edge formation in unweighted attributed multigraphs. To that end, we propose intuitive models for edge formation (Section “Generative edge formation models”), a flexible toolbox to formally specify belief in the model parameters (Section “Constructing belief matrices”), a way of computing proper (Dirichlet) priors from these beliefs (Section “Eliciting a Dirichlet prior.”), computation of the marginal likelihood in this scenario (Section “Computation of the marginal likelihood”), and guidelines on how to interpret the results (Section “Application of the method and interpretation of results”). We subsequently discuss these issues one-by-one.

### Generative edge formation models

We propose two variations of our approach, which employ two different types of generative edge formation models in multigraphs.

**Fig. 2:**
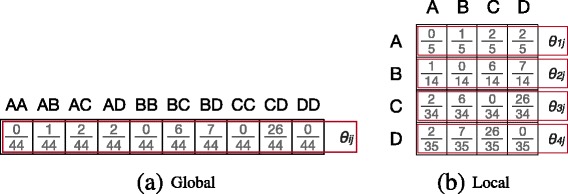
Multigraph models: This figure shows two ways of modeling the undirected multigraph shown in Fig. 1. That is, **a** global or graph-based model models the whole graph as a single distribution. **b** Local or neighbour-based model models each node as a separate distribution

**Global model** First, we utilize a simple *global model*, in which a fixed number of graph edges are randomly and independently drawn from the set of all potential edges in the graph *G* by sampling with replacement. Each edge (*v*_*i*_,*v*_*j*_) is sampled from a *categorical distribution* with parameters $\theta _{ij}, 1 \leq i \leq n, 1 \leq j \leq n, \forall {ij}: \sum _{ij} \theta _{ij} = 1$: (*v*_*i*_,*v*_*j*_)∼*Categorical*(*θ*_*ij*_). This means that each edge is associated with one probability *θ*_*ij*_ of being drawn next. Figure 2 a shows the maximum likelihood global model for the network shown in Fig. 1. Since this is an undirected graph, inverse edges can be ignored resulting in *n*(*n*+1)/2 potential edges/parameters.

**Local models** As an alternative, we can also focus on a *local level*. Here, we model to which other node a specific node *v* will connect *given that any new edge starting from v* is formed. We implement this by using a set of *n* separate models for the outgoing edges of the ego-networks (i.e., the 1-hop neighborhood) of each of the *n* nodes. The ego-network model for node *v*_*i*_ is built by drawing randomly and independently a number of nodes *v*_*j*_ by sampling with replacement and adding an edge from *v*_*i*_ to this node. Each node *v*_*j*_ is sampled from a *categorical distribution* with parameters $\theta _{ij}, 1 \leq i \leq n, 1 \leq j \leq n, \forall i: \sum _{j} \theta _{ij} = 1$: *v*_*j*_∼*Categorical*(*θ*_*ij*_). The parameters *θ*_*ij*_ can be written as a matrix; the value in cell (*i,j*) specifies the probability that a new formed edge with source node *v*_*i*_ will have the destination node *v*_*j*_. Thus, all values within one row always sum up to one. Local models can be applied for undirected and directed graphs (cf. also in Section “Discussion”). In the directed case, we model only the outgoing edges of the ego-network. Figure 2 b depicts the maximum likelihood local models for our introductory example.

### Hypothesis elicitation

The main idea of our approach is to encode our beliefs in edge formation as Bayesian priors over the model parameters. As a common choice, we employ Dirichlet distributions as the *conjugate priors* of the categorical distribution. Thus, we assume that the model parameters *θ* are drawn from a Dirichlet distribution with hyperparameters *α*: *θ*∼*Dir*(*α*). Similar to the model parameters themselves, the Dirichlet prior (or multiple priors for the local models) can be specified in a matrix. We will choose the parameters *α* in such a way that they reflect a specific belief about edge formation. For that purpose, we first specify matrices that formalize these beliefs, then we compute the Dirichlet parameters *α* from these beliefs.

#### Constructing belief matrices

We specify hypotheses about edge formation as *belief matrices* *B*=*b*_*ij*_. These are *n*×*n* matrices, in which each cell *b*_*ij*_∈IR represents a belief of having an edge from node *v*_*i*_ to node *v*_*j*_. To express a belief that an edge occurs more often (compared to other edges) we set *b*_*ij*_ to a higher value.

**Node-attributed multigraphs** In general, users have a large freedom to generate belief matrices. However, typical construction principles are to assume that nodes with specific attributes are more *popular* and thus edges connecting these attributes receive higher multiplicity, or to assume that nodes that are *similar* with respect to one or more attributes are more likely to form an edge, cf. (Papadopoulos et al. 2012). Ideally, the elicitation of belief matrices is based on existing theories.

For example, based on the information shown in Fig. 1, one could “believe” that two authors collaborate *more frequently* together if: (1) they both are from the same country, (2) they share the same gender, (3) they have high positions, or (4) they are popular in terms of number of articles and citations. We capture each of these beliefs in one matrix. One implementation of the matrices for our example beliefs could be:

- *B*_1_ (same country): *b*_*ij*_:=0.9 if *f*_*i*_[*country*]=*f*_*j*_[*country*] and 0.1 otherwise
- *B*_2_ (same gender): *b*_*ij*_:=0.9 if *f*_*i*_[*gender*]=*f*_*j*_[*gender*] and 0.1 otherwise
- *B*_3_ (hierarchy): *b*_*ij*_:=*f*_*i*_[*position*]·*f*_*j*_[*position*]
- *B*_4_ (popularity): *b*_*ij*_:=*f*_*i*_[*articles*]+*f*_*j*_[*articles*]+*f*_*i*_[*citations*]+*f*_*j*_[*citations*]

**Fig. 3:**
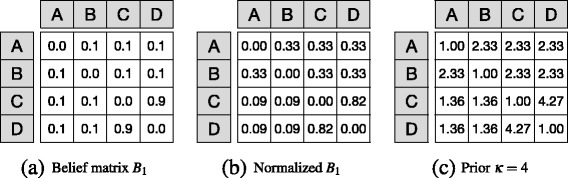
Prior belief: This figure illustrates the three main phases of prior elicitation. That is, **a** a matrix representation of belief *B*_1_, where authors are more likely to collaborate with each other if they are from the same country. **b** *B*_1_ normalized row-wise using the local model interpretation. **c** Prior elicitation for *κ*=4; i.e., $\alpha _{ij} = \frac {b_{ij}}{Z} \times \kappa + 1$

Figure 3 a shows the matrix representation of belief *B*_1_, and Fig. 3 b its respective row-wise normalization for the local model case. While belief matrices are identically structured for local and global models, the ratio between parameters in different rows is crucial for the global model, but irrelevant for local ones.

**Dyad-attributed networks** For the particular case of Dyad-Attributed networks, beliefs are described using the underlying mechanisms of secondary multigraphs. For instance, a *co-authorship* network—where every node represents an author with no additional information or attribute—could be explained by a *citation* network under the hypothesis that if two authors frequently cite each other, they are more likely to also co-author together. Thus, the adjacency (feature) matrices $(\hat F)$ of secondary multigraphs can be directly used as belief matrices *B*=(*b*_*ij*_). However, we can express additional beliefs by transforming the matrices. As an example, we can formalize the belief that the presence of a feature tends to inhibit the formation of edges in the data by setting *b*_*ij*_:=−*sigm*(*f*_*ij*_), where *sigm* is a sigmoid function such as the logistic function.

#### Eliciting a Dirichlet prior.

In order to obtain the hyperparameters *α* of a prior Dirichlet distribution, we utilize the pseudo-count interpretation of the parameters *α*_*ij*_ of the Dirichlet distribution, i.e., a value of *α*_*ij*_ can be interpreted as *α*_*ij*_−1 previous observations of the respective event for *α*_*ij*_≥1. We distribute pseudo-counts proportionally to a belief matrix. Consequently, the hyperparameters can be expressed as: $\alpha _{ij} = \frac {b_{ij}}{Z} \times \kappa + 1$, where *κ* is the concentration parameter of the prior. The normalization constant *Z* is computed as the sum of all entries of the belief matrix in the global model, and as the respective row sum in the local case. We suggest to set *κ*=*n*×*k* for the local models, *κ*=*n* ^2^×*k* for the directed global case, $\kappa = \frac {n(n+1)}{2} \times k$ for the undirected global case, and *k*={0,1,...,10}. A high value of *κ* expresses a strong belief in the prior parameters. A similar alternative method to obtain Dirichlet priors is the *trial roulette method* (Singer et al. 2015). For the global model variation, all *α* values are parameters for the same Dirichlet distribution, whereas in the local model variation, each row parametrizes a separate Dirichlet distribution. Figure 3 (c) shows the prior elicitation of belief *B*1 for *kappa*=4 using the local model.

### Computation of the marginal likelihood

For comparing the relative plausibility of hypotheses, we use the marginal likelihood. This is the aggregated likelihood over all possible values of the parameters *θ* weighted by the Dirichlet prior. For our set of local models we can calculate them as:

2 $$ P(D|H) = \prod_{i=1}^{n} \frac{\Gamma\left({\sum\nolimits}_{j=1}^{n}\alpha_{ij}\right)}{\Gamma\left({\sum\nolimits}_{j=1}^{n}\alpha_{ij}+m_{ij}\right)} \prod_{j=1}^{n} \frac{\Gamma(\alpha_{ij}+m_{ij})}{\Gamma(\alpha_{ij})}  $$

Recall, *α*_*ij*_ encodes our prior belief connecting nodes *v*_*i*_ and *v*_*j*_ in *G*, and *m*_*ij*_ are the actual edge counts. Since we evaluate only a single model in the global case, the product over rows *i* of the adjacency matrix can be removed, and we obtain:

3 $$ P(D|H)=\frac{\Gamma\left({\sum\nolimits}_{i=1}^{n}{\sum\nolimits}_{j=1}^{n}\alpha_{ij}\right)}{\Gamma\left({\sum\nolimits}_{i=1}^{n}{\sum\nolimits}_{j=1}^{n}\alpha_{ij}+m_{ij}\right)} \prod_{i=1}^{n}\prod_{j=1}^{n} \frac{\Gamma\left(\alpha_{ij}+m_{ij}\right)}{\Gamma(\alpha_{ij})}  $$

Section “Computation of the marginal likelihood” holds for directed networks. In the undirected case, indices *j* go from *i* to *n* accounting for only half of the matrix including the diagonal to avoid inconsistencies. For a detailed derivation of the marginal likelihood given a Dirichlet-Categorical model see (Tu 2014; Singer et al. 2014). For both models we focus on the log-marginal likelihoods in practice to avoid underflows.

**Bayes factor** Formally, we compare the relative plausibility of hypotheses by using so-called *Bayes factors* (Kass and Raftery 1995), which simply are the ratios of the marginal likelihoods for two hypotheses *H*_1_ and *H*_2_. If it is positive, the first hypothesis is judged as more plausible. The strength of the Bayes factor can be checked in an interpretation table provided by Kass and Raftery (1995).

### Application of the method and interpretation of results

We now showcase an example application of our approach featuring the network shown in Fig. 1, and demonstrate how results can be interpreted.

**Hypotheses** We compare four hypotheses (represented as belief matrices) *B*_1_, *B*_2_, *B*_3_, and *B*_4_ elaborated in Section “Hypothesis elicitation”. Additionally, we use the *uniform* hypothesis as a *baseline*. It assumes that all edges are equally likely, i.e., *b*_*ij*_=1 for all *i,j*. Hypotheses that are not more plausible than the uniform cannot be assumed to capture relevant underlying mechanisms of edge formation. We also use the *data* hypothesis as an upper bound for comparison, which employs the observed adjacency matrix as belief: *b*_*ij*_=*m*_*ij*_.

**Fig. 4:**
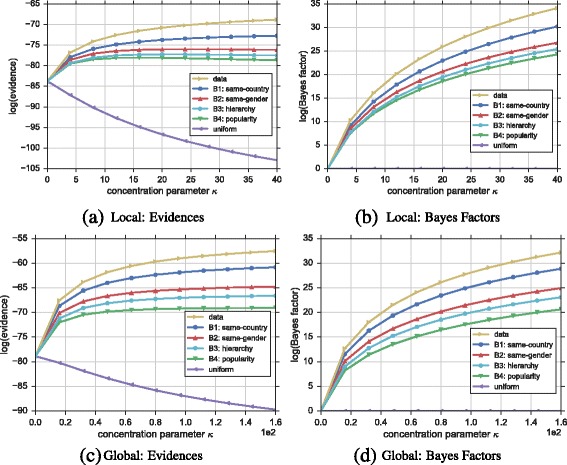
Ranking of hypotheses for the introductory example. **a**, **b** Represent results using the local model and **c**, **d** results of the global model. Rankings can be visualized using **a**, **c** the marginal likelihood or evidence (y-axis), or **b**, **d** using Bayes factors (y-axis) by setting the uniform hypothesis as a baseline to compare with; higher values refer to higher plausibility. The x-axis depicts the concentration parameter *κ*. For this example, from an individual perspective (local model) authors from the multigraph shown in Fig. 1 appear to prefer to collaborate more often with researchers of the same country rather than due to popularity (i.e., number of articles and citations). In this particular case, the same holds for the global model. Note that all hypotheses outperform the uniform, meaning that they all are reasonable explanations of edge formation for the given graph

**Calculation and visualization** For each hypothesis *H* and every *κ*, we can elicit the Dirichlet priors (cf. Section “Hypothesis elicitation”), determine the aggregated marginal likelihood (cf. Section “Computation of the marginal likelihood)”, and compare the plausibility of hypotheses compared to the uniform hypothesis at the same *κ* by calculating the logarithm of the Bayes factor as *log*(*P*(*D*|*H*))−*log*(*P*(*D*|*H*_*uniform*_)). We suggest two ways of visualizing the results, i.e., plotting the marginal likelihood values, and showing the Bayes factors on the y-axis as shown in Fig. 4 a and 4 b respectively for the local model. In both cases, the x-axis refers to the concentration parameter *κ*. While the visualization showing directly the marginal likelihoods carries more information, visualizing Bayes factors makes it easier to spot smaller differences between the hypotheses.

**Interpretation** Every line in Fig. 4 a to 4 d represents a hypothesis using the local (top) and global models (bottom). In Fig. 4 a and 4 c, higher evidence values mean higher plausibility. Similarly, in Fig. 4 b and 4 d positive Bayes factors mean that for a given *κ*, the hypothesis is judged to be more plausible than the uniform baseline hypothesis; here, the relative Bayes factors also provide a ranking. If evidences or Bayes factors are increasing with *κ*, we can interpret this as further evidence for the plausibility of expressed hypothesis as this means that the more we believe in it, the higher the Bayesian approach judges its plausibility. As a result for our example, we see that the hypothesis believing that two authors are more likely to collaborate if they are from the same country is the most plausible one (after the data hypothesis). In this example, all hypotheses appear to be more plausible than the baseline in both local and global models, but this is not necessarily the case in all applications.

## Experiments

We demonstrate the utility of our approach on both synthetic and empirical networks.

### Synthetic node-attributed multigraph

We start with experiments on a synthetic node-attributed multigraph. Here, we control the underlying mechanisms of how edges in the network emerge and thus, expect these also to be good hypotheses for our approach.

**Fig. 5:**
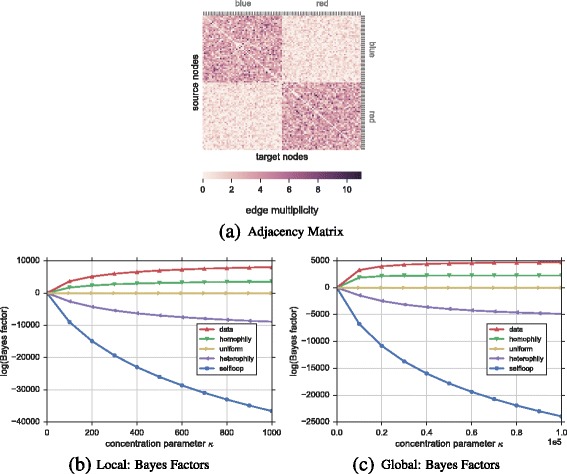
Ranking of hypotheses for synthetic attributed multigraph. In **a**, we show the adjacency matrix of a 100-node 2-color random multigraph with a node correlation of 80*%* for nodes of the same color and 20*%* otherwise. One can see the presence of homophily based on more connections between nodes of the same color; the diagonal is zero as there are no self-connections. In **b**, **c** we show the ranking of hypotheses based on Bayes factors when compared to the uniform hypothesis for the local and global models respectively. As expected, in general the homophily hypothesis explains the edge formation best (positive Bayes factor and close to the data curve), while the heterophily and selfloop hypotheses provide no good explanations for edge formation in both local and global cases—they show negative Bayes factors

**Network** The network contains 100 nodes where each node is assigned one of two colors with uniform probability. For each node, we then randomly drew 200 undirected edges where each edge connects randomly with probability *p*=0.8 to a different node of the same color, and with *p*=0.2 to a node of the opposite color. The adjacency matrix of this graph is visualized in Fig. 5 a.

**Hypotheses** In addition to the uniform baseline hypothesis, we construct two intuitive hypotheses based on the node color that express belief in possible edge formation mechanics. First, the *homophily* hypothesis assumes that nodes of the same color are more likely to have more edges between them. Therefore, we arbitrary set belief values *b*_*ij*_ to 80 when nodes *v*_*i*_ and *v*_*j*_ are of the same color, and 20 otherwise. Second, the *heterophily* hypothesis expresses the opposite behavior; i.e., *b*_*ij*_=80 if the color of nodes *v*_*i*_ and *v*_*j*_ are different, and 20 otherwise. An additional *selfloop* hypothesis only believes in self-connections (i.e., diagonal of adjacency matrix).

**Results** Figure 5 b and 5 c show the ranking of hypotheses based on their Bayes factors compared to the uniform hypothesis for the local and global models respectively. Clearly, in both models the homophily hypothesis is judged as the most plausible. This is expected and corroborates the fact that network connections are biased towards nodes of the same color. The heterophily and selfloop hypotheses show negative Bayes factors; thus, they are not good hypotheses about edge formation in this network. Due to the fact that the multigraph lacks of selfloops, the selfloop hypothesis decreases very quickly with increasing strength of belief *κ*.

### Synthetic multiplex network

In this experiment, we control the underlying mechanisms of how edges in a dyad-attributed multigraph emerge using multiple multigraphs that share the same nodes with different link structure (i.e., multiplex) and thus, expect these also to be good hypotheses for JANUS.

**Fig. 6:**
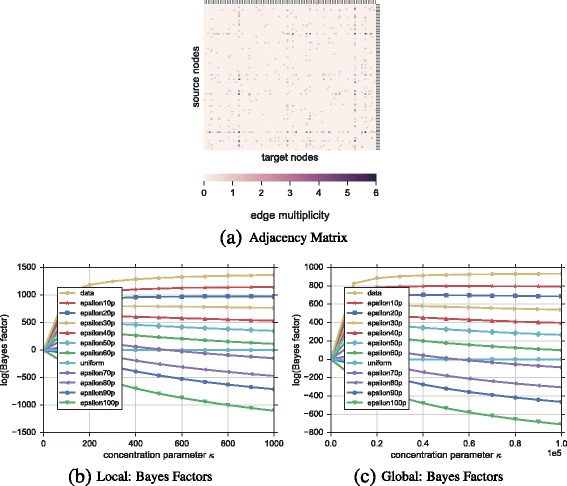
Ranking of hypotheses for synthetic multiplex network. In **a** we show the adjacency matrix of a configuration model graph of 100 nodes and power-law distributed degree sequence. In **b**, **c** the ranking of hypotheses is shown for the local and global model respectively. As expected, hypotheses are ranked from small to big values of *ε* since small values represent only a few changes in the original adjacency matrix of the configuration graph. Both models show that when the original graph changes at least 70*%* of its edges the new graph cannot be explained better than random (i.e., uniform)

**Network** The network is an undirected *configuration model* graph (Newman 2003) with parameters *n*=100 (i.e., number of nodes) and degree sequence $\overrightarrow {k}={k_{i}}$ drawn from a power law distribution of length *n* and exponent 2.0, where *k*_*i*_ is the degree of node *v*_*i*_. The adjacency matrix of this graph is visualized in Fig. 6 a.

**Hypotheses** Besides the uniform hypothesis, we include ten more hypotheses derived from the original adjacency matrix of the configuration model graph where only certain percentage *ε* of edges get shuffled. The bigger the *ε* the less plausible the hypothesis since more shuffles can modify drastically the original network.

**Results** Figure 6 b and 6 c show the ranking of hypotheses based on their Bayes factors compared to the uniform hypothesis for the local and global model respectively. In general, hypotheses are ranked as expected, from small to big values of *ε*. For instance, the *epsilon10p* hypothesis explains best the *configuration model* graph—represented in Fig. 6 a—since it only shuffles 10*%* of all edges (i.e., 10 edges). On the other hand the *epsilon100p* hypothesis shows the worst performance (i.e., Bayes factor is negative and far from the data curve) since it shuffles all edges, therefore it is more likely to be different than the original network.

### Empirical node-attributed multigraph

Here, we focus on a real-world contact network based on wearable sensors.

**Fig. 7:**
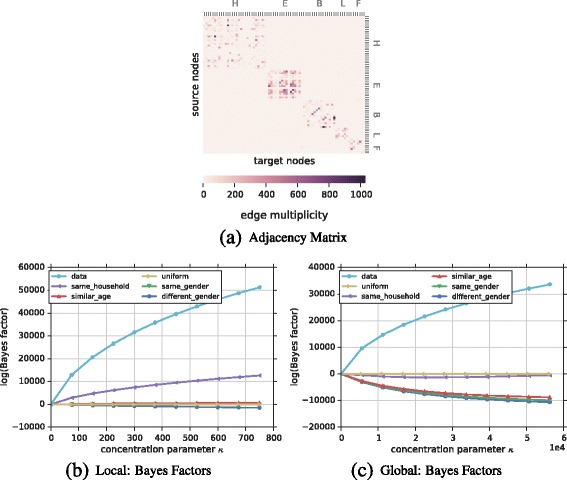
Ranking of hypotheses for Kenya contact network. **a** Shows the adjacency matrix of the network with node ordering according to household membership. Darker cells indicate more contacts. **b**, **c** Display the ranking of hypotheses based on Bayes factors, using the uniform hypothesis as baseline for the local and global model respectively. Using the local model **b** the *same household* hypothesis ranks highest followed by the *similar age* hypothesis which also provides positive Bayes Factors. On the other hand, the *same* and *different gender* hypotheses are less plausible than the baseline (uniform edge formation) in both the local and global case. In the global case **c** all hypotheses are bad representations of edge formation in the Kenya contact network. This is due to the fact that interactions are very sparse, even within households. Results are consistent for all *κ*

**Network** We study a network capturing interactions of 5 households in rural Kenya between April 24 and May 12, 2012 (Sociopatterns; Kiti et al. 2016). The undirected unweighted multigraph contains 75 nodes (persons) and 32, 643 multiedges (contacts) which we aim to explain. For each node, we know information such as gender and age (encoded into 5 age intervals). Interactions exist within and across households. Figure 7 a shows the adjacency matrix (i.e., number of contacts between two people) of the network. Household membership of nodes (rows/columns) is shown accordingly.

**Hypotheses** We investigate edge formation by comparing—next to the uniform baseline hypothesis—four hypotheses based on node attributes as prior beliefs. (i) The *similar age* hypothesis expresses the belief that people of similar age are more likely to interact with each other. Entries *b*_*ij*_ of the belief matrix *B* are set to the inverse age distance between members: $\frac {1}{1+abs(f_{i}[age]-f_{j}[age])}$. (ii) The *same household* hypothesis believes that people are more likely to interact with people from the same household. We arbitrarily set *b*_*ij*_ to 80 if person *v*_*i*_ and person *v*_*j*_ belong to the same household, and 20 otherwise. (iii) With the *same gender* hypothesis we hypothesize that the number of same-gender interactions is higher than the different-gender interactions. Therefore, every entry *b*_*ij*_ of *B* is set to 80 if persons *v*_*i*_ and *v*_*j*_ are of the same gender, and 20 otherwise. Finally, (iv) the *different gender* hypothesis believes that it is more likely to find different-gender than same-gender interactions; *b*_*ij*_ is set to 80 if person *v*_*i*_ has the opposite gender of person *v*_*j*_, and 20 otherwise.

**Results** Results shown in Fig. 7 b and 7 c show the ranking of hypotheses based on Bayes factors using the uniform hypothesis as baseline for the local and global model respectively. The local model Fig. 7 b indicates that the *same household* hypothesis explains the data the best, since it has been ranked first and it is more plausible than the uniform. The *similar age* hypothesis also indicates plausibility due to positive Bayes factors. Both the *same* and *different gender* hypotheses show negative Bayes factors when compared to the uniform hypothesis suggesting that they are not good explanations of edge formation in this network. This gives us a better understanding of potential mechanisms producing underlying edges. People prefer to contact people from the same household and similar age, but not based on gender preferences. Additional experiments could further refine these hypotheses (e.g., combining them). In the general case of the global model in Fig. 7 c all hypotheses are bad explanations of the Kenya network. However, the *same-household* hypothesis tends to go upfront the uniform for higher values of *κ*, but still far form the data curve. This happens due to the fact that the interaction network is very sparse (even within same households), thus, any hypothesis with a dense belief matrix will likely fall below or very close to the uniform.

### Empirical multiplex network

This empirical dataset consists of four real-world social networks, each of them extracted from Twitter interactions of a particular set of users.

**Network** We obtained the Higgs Twitter dataset from SNAP (SNAP Higgs Twitter datasets). This dataset was built upon the interactions of users regarding the discovery of a new particle with the features of the elusive Higgs boson on the 4th of July 2012 (De Domenico et al. 2013). Specifically, we are interested on characterizing edge formation in the *reply network*, a directed unweighted multigraph which encodes the replies that a person *v*_*i*_ sent to a person *v*_*j*_ during the event. This graph contains 38, 918 nodes and 36, 902 multiedges (if all edges from the same dyad are merged it accounts for 32, 523 weighted edges).

**Hypotheses** We aim to characterize the reply network by incorporating other networks—sharing the same nodes but different network structure—as prior beliefs. In this way we can learn whether the interactions present in the reply network can be better explained by a retweet or mentioning or following (social) network. The *retweet* hypothesis expresses our belief that the number of replies is proportional to the number of retweets. Hence, beliefs *b*_*ij*_ are set to the number of times user *v*_*i*_ retweeted a post from user *v*_*j*_. Similar as before, the *mention* hypothesis states that the number of replies is proportional to the number of mentions. Therefore, every entry *b*_*ij*_ is set to the number of times user *v*_*i*_ mentioned user *v*_*j*_ during the event. The *social* hypothesis captures our belief that users are more likely to reply to their friends (in the Twitter jargon: followees or people they follow) than to the rest of users. Thus, we set *b*_*ij*_ to 1 if user *v*_*i*_ follows user *v*_*j*_ and 0 otherwise. Finally, we combine all the above networks to construct the *retweet-mention-social* hypothesis which captures all previous hypotheses at once. In other words, it reflects our belief that users are more likely to reply to their friends and (at the same time) the number of replies is proportional to the number of retweets and mentions. Therefore the adjacency matrix for this hypothesis is simply the sum of the three networks described above.

**Fig. 8:**
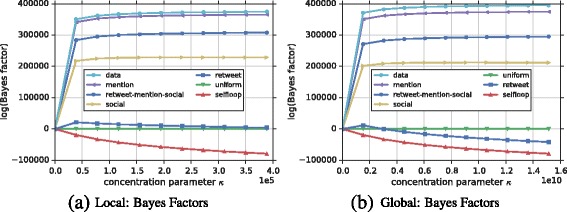
Ranking of hypotheses for Reply Higgs Network. **a**, **b** Ranking of hypotheses based on Bayes factors when compared to the uniform hypothesis using multiplexes for the local and global models respectively. In both cases, the *mention* hypothesis explains best the reply network, since it is ranked first and very close to the data curve. This might be due to the fact that replies inherit a user mention from whom a tweet was originally posted. We can see that the combined *retweet-mention-social* hypothesis is the second best explanation of the reply network. This is mainly due to the mention hypothesis which performs extremely better than the other two (social and retweet). The *social* hypothesis can also be considered a good explanation since it outperforms the uniform. The *retweet* hypothesis tends to perform worse than the uniform in both cases for increasing number of *κ*. Similarly, the *selfloop* hypothesis drops down below the uniform since there are only very few selfloops in the *reply* network data

**Results** The results shown in Fig. 8 suggest that the *mention* hypothesis explains the reply network very well, since it has been ranked first and it is very close to the data curve, in both Fig. 8 a and 8 b for the local and global models, respectively. The *retweet-mention-social* hypothesis also indicates plausibility since it outperforms the uniform (i.e., positive Bayes factors). However, if we look at each hypothesis individually, we can see that the combined hypothesis is dominated mainly by the *mention* hypothesis. The *social* hypothesis is also a good explanation of the number of replies since it outperforms the uniform hypothesis. *Retweets* and *Self-loops* on the other hand show negative Bayes factors, suggesting that they are not good explanations of edge formation in the reply network. Note that the retweet curve in the local model has a very strong tendency to go below the uniform for higher numbers of *κ*. These results suggest us that the number of replies is proportional to the number of mentions and that usually people prefer to reply other users within their social network (i.e., followees).

## Discussion

Next, we discuss some aspects and open questions related to the proposed approach.

**Comparison to existing method** While we have already demonstrated the plausibility of JANUS based on synthetic datasets, we want to discuss how our results compare to existing state-of-the-art methods. A simple alternative approach to evaluate the plausibility of beliefs as expressed by the belief matrices is to compute a Pearson correlation coefficient between the entries in the belief matrix and the respective entries in the adjacency matrix of the network. To circumvent the difficulties of correlating matrices, they can be flattened to vectors that are then passed to the correlation calculation. Then, hypotheses can be ranked according to their resulting correlation against the data. However, by flattening the matrices, we disregard the direct relationship between nodes in the matrix and introduce inherent dependencies to the individual data points of the vectors used for Pearson calculation. To tackle this issue, one can utilize the Quadratic Assignment Procedure (QAP) as mentioned in Section “Related work”. QAP is a widely used technique for testing hypotheses on dyadic data (e.g., social networks). It extends the simple Pearson correlation calculation step by a significance test accounting for the underlying link structure in the given network using shuffling techniques. For a comparison with our approach, we executed QAP for all datasets and hypotheses presented in Section “Experiments” using the qaptest function included in the statnet (Handcock et al. 2008; Handcock et al. 2016) package in R (R Core Team 2016).

Overall, we find in all experiments strong similarities between the ranking provided by the correlation coefficients of QAP and our rankings according to JANUS. Exemplary, Table 1 shows the correlation coefficients and p-values obtained with QAP for each hypothesis tested on the synthetic multiplex described in Section “Synthetic multiplex network” as well as the ranking of hypotheses obtained from JANUS for the local and global model (leaving the uniform hypothesis out). However, in other datasets minor differences in the ordering of the hypotheses could be observed between the two approaches.

**Table 1:** QAP on synthetic dyad-attributed network (multiplex): List of correlation coefficients for each hypothesis tested. Last two columns show ranking of hypotheses according to JANUS for the local and global models. By omitting the uniform hypothesis in JANUS (rank 7) we can see that the ranking of hypotheses by correlation aligns with the rankings given by JANUS for the multiplex given in Section “Synthetic multiplex network”

Hypothesis	Correlation Coefficient	*P-Value*	JANUS Ranking Local	JANUS Ranking Global
Epsilon10p	0.939	0.0**	1	1
Epsilon20p	0.863	0.0**	2	2
Epsilon30p	0.787	0.0**	3	3
Epsilon40p	0.704	0.0**	4	4
Epsilon50p	0.636	0.0**	5	5
Epsilon60p	0.461	0.0**	6	6
Epsilon70p	0.352	0.0**	8	8
Epsilon80p	0.242	0.0**	9	9
Epsilon90p	0.142	0.0**	10	10
Epsilon100p	0.010	0.238	11	11

Statistically highly significant *p*-values (*p*<0.001) are marked by (**)

Compared to QAP, JANUS yields several advantages, but also some disadvantages. First, by utilizing our belief matrix as priors over parameter configurations instead of fixed parameter configurations themselves, we allow for tolerance in the parameter specification. Exploring different values of tolerance expressed by our parameter *κ* allows for more fine-grained and advanced insights into the relative plausibility of hypotheses. Contrary, simple correlation takes the hypothesis as it is and calculates a single correlation coefficient that does not allow for tolerances.

Second, by building upon Bayesian statistics, the significance (or decisiveness) of results in our approach is determined by Bayes factors, a Bayesian alternative to traditional p-value testing. Instead of just measuring evidence *against* one null hypothesis, Bayes Factors allow to directly gather evidence *in favor* of a hypothesis compared to another hypothesis, which is arguably more suitable for ranking.

Third, QAP and MRQAP, and subsequently correlation and regression, are subject to multiple assumptions which our generative Bayesian approach circumvents. Currently, we employ QAP with simplistic linear Pearson correlation coefficients. However, one could argue that count data (multiplicity of edges) warrants advanced generalized linear models such as Poisson regression or Negative Binomial regression models.

Furthermore, our approach intuitively allows to model not only the overall network, but also the ego-networks of the individual nodes using the local models presented above. Finally, correlation coefficients cannot be applied for all hypotheses. Specifically, it is not possible to compute it for the uniform hypothesis since in this case all values in the flatten vector are identical. However, our method currently does not sufficiently account for dependencies within the network as it is done by specialized QAP significance tests. Exploring this issue and extending our Bayesian approach into this direction will be a key subject of future work.

**Runtime performance** A typical concern often associated with Bayesian procedures are the excessive runtime requirements, especially if calculating marginal likelihoods is necessary. However, the network models employed for this paper allow to calculate the marginal likelihoods—and consequently also the Bayes factors—efficiently in closed form. This results in runtimes, which are not only competitive with alternative methods such as QAP and MRQAP, but could be calculated up to 400 times faster than MRQAP in our experiments as MRQAP requires many data reshuffles and regression fits. Furthermore, the calculation (of Bayesian evidence) could easily be distributed onto several computational units, cf. (Becker et al. 2016).

**Local vs global model** In this paper, we presented two variations of our approach, i.e., a local and a global model. Although both model substantially different generation processes (an entire network vs. a set of ego-networks), our experiments have shown that hypotheses in the global scenario are ranked mostly the same as the ones using the local model. This is also to be expected to some degree since the constructed hypotheses did not explicitly expressed a belief that outgoing links are more likely for some nodes.

**Inconsistency of local model** For directed networks, the local ego-network models can assemble a full graph model by defining a probability distribution of edges for every source node. For undirected networks, this is not directly possible as e.g., the ego-network model for *v*_*A*_ generated an edge from *v*_*A*_ to *v*_*B*_, but the ego-network model for node *v*_*B*_ did not generate any edge to *v*_*A*_. Note that this does not affect our comparison of hypotheses as we characterize the network.

**Single Edges** As mentioned in Section “Background”, JANUS focuses on multigraphs, meaning that edges might appear more than once. This is because we assume that a given node *v*_*i*_, with some probability *p*_*ij*_, will be connected *multiple* times to any other node *v*_*j*_ in the local models. The same applies to the global model where we assume that a given edge (*v*_*i*_,*v*_*j*_) will appear *multiple* times within the graph with some probability *p*_*ij*_. For the specific case of single edges (i.e., unweighted graphs), where *m*_*ij*_∈{0,1}, one might consider other probabilistic models to represent such graphs.

**Sparse data-connections** Most real networks exhibit small world properties such as high clustering coefficient and fat-tailed degree distributions meaning that the adjacency matrices are sparse. While comparison still relatively judges the plausibility, all hypotheses perform weak compared to the data curve as shown in Fig. 7. As an alternative, one might want to limit our beliefs to only those edges that exist in the network, i.e., we would then only build hypotheses on how edge multiplicity varies between edges.

**Other limitations and future work** The main intent of this work is the introduction of a hypothesis-driven Bayesian approach for understanding edge formation in networks. To that end, we showcased this approach on simple categorical models that warrant extensions, e.g., by incorporating appropriate models for other types of networks such as weighted or temporal networks. We can further investigate how to build good hypotheses by leveraging all node attributes, and infer subnetworks that fit best each of the given hypotheses. In the future, we also plan an extensive comparison to other methods such as mixed-effects models and *p* ^∗^ models. Ultimately, our models also warrant extensions to adhere to the degree sequence in the network, e.g., in the direction of multivariate hypergeometric distributions as recently proposed in (Casiraghi et al. 2016).

## Conclusions

In this paper, we have presented a Bayesian framework that facilitates the understanding of edge formation in node-attributed and dyad-attributed multigraphs. The main idea is based on expressing hypotheses as beliefs in parameters (i.e., multiplicity of edges), incorporate them as priors, and utilize Bayes factors for comparing their plausibility. We proposed simple local and global Dirichlet-categorical models and showcased their utility on synthetic and empirical data. For illustration purposes our examples are based on small networks. We tested our approach with larger networks obtaining identical results. We briefly compare JANUS with existing methods and discuss some advantages and disadvantages over the state-of-the-art QAP. In future, our concepts can be extended to further models such as models adhering to fixed degree sequences. We hope that our work contributes new ideas to the research line of understanding edge formation in complex networks.
